# Non-compartment model to compartment model pharmacokinetics transformation meta-analysis – a multivariate nonlinear mixed model

**DOI:** 10.1186/1752-0509-4-S1-S8

**Published:** 2010-05-28

**Authors:** Zhiping Wang, Seongho Kim, Sara K Quinney, Jihao Zhou, Lang Li

**Affiliations:** 1 Division of Biostatistics, Department of Medicine, School of Medicine, Indiana University, Indianapolis, IN, 46032, USA; 2 Department of Biostatistics, School of Public Health, University of Michigan, Ann Arbor, MI, 48109, USA

## Abstract

**Background:**

To fulfill the model based drug development, the very first step is usually a model establishment from published literatures. Pharmacokinetics model is the central piece of model based drug development. This paper proposed an important approach to transform published non-compartment model pharmacokinetics (PK) parameters into compartment model PK parameters. This meta-analysis was performed with a multivariate nonlinear mixed model. A conditional first-order linearization approach was developed for statistical estimation and inference.

**Results:**

Using MDZ as an example, we showed that this approach successfully transformed 6 non-compartment model PK parameters from 10 publications into 5 compartment model PK parameters. In simulation studies, we showed that this multivariate nonlinear mixed model had little relative bias (<1%) in estimating compartment model PK parameters if all non-compartment PK parameters were reported in every study. If there missing non-compartment PK parameters existed in some published literatures, the relative bias of compartment model PK parameter was still small (<3%). The 95% coverage probabilities of these PK parameter estimates were above 85%.

**Conclusions:**

This non-compartment model PK parameter transformation into compartment model meta-analysis approach possesses valid statistical inference. It can be routinely used for model based drug development.

## Background

In recent decades, a new drug requires an average of 15 years and approaching a billion dollars in research and development [1]. Unfortunately, only one in 10 drugs that enter clinical testing receives eventual FDA approval [2,3]. Scientists have become increasingly mechanistic in their approach to drug development [4]. The recent ability to integrate genetic mutations and altered protein expression to pharmacokinetics (PK) and pharmacodynamic (PD) models allow a deeper understanding of the mechanisms of disease and therapies that are genuinely targeted [5-8]. In 2004, the FDA released a report entitled: “Innovation or Stagnation, Challenge and Opportunity on the Critical Path to New Medical Products” [9]. Among its six general topic areas, three of them emphasized the importance of computational modeling and bioinformatics in biomarker development and streamlining clinical trials [10,11]. In multiple follow-up papers, clinical researchers, experimental biologists, computational biologists, and biostatisticians from both academia and industry all supported the FDA leadership in this critical path, and pointed out the challenges and opportunities of the PK/PD model based approach in drug development [12][13-15].

Pharmacokinetics model is the central piece of model based drug development. Almost all of the published PK data were summarized without fitting a compartment model. They are usually called non-compartment model PK parameters. For example, area under the concentration curve (AUC) is calculated from drug plasma concentration data based on trapezoid-rule [16]; clearance is calculated from dose and AUC; Cmax and Tmax are calculated from concentrations and their associated time points; terminal half-life is usually calculated from the last two to four sampling time-points directly; and etc. All these parameters cannot be used directly in a compartment model, and their transformation to compartment model PK parameters is essential.

## Methods

### Non-Compartment Model to One-Compartment Model Transformation

When a drug follows a one-compartment model of oral dose (1), the following non-compartment model PK parameters, **w** = (*AUC, T_max,_ T_1/2_*), are necessary to recover the one-compartment model parameters, β = (*k_a,_ k_e,_ V*).

				(1)

		(2)

where, *F* is an assumed known bioavailability, and dose denotes the oral dose. If however, only oral clearance, *CL_po_* is reported, instead of *AUC*, then *CL _po_ = V × K_e_*. On the other hand, when dosing is through IV, only **w** = (*AUC, T_1/2_*), are necessary to recover the one compartment (3), with **β** = (*k_e,_ V*). The transformation formulas are defined in (4).

						(3)

						(4)

Similarly, if *CL_iv_* is reported, instead of *AUC*, then *CL_IV_ = V × k_e_*. These one-compartment-model and non-compartment model parameters and transformation were defined and discussed in great detail by [16].

### Non-Compartment Model to Two-Compartment Model Transformation

If a drug’s pharmacokinetics follows a two-compartment model with oral dose (5), the following non-compartment model PK parameters, **w** = (*Vd*, *AUC, T_max,_ CL_iv,_ T_1/2,slow,_ T_1/2,fast_*), are necessary to recover the two-compartment model parameters, **β** = (*k_a,_ k_e,_ V_1,_ k_12,_ k_21_*). Their transformations are defined in (6).

		(5)

			(6)

If a drug’s pharmacokinetics follows a two-compartment model with IV dose (7), the following non-compartment model PK parameters, **w** = (*Vd*, *AUC, CL_iv,_ T_1/2, slow,_ T_1/2, fast_*), are necessary to recover the two-compartment model parameters, **β** = (*k_e,_V_1,_ k_12,_ k_21_*). Their transformations are defined in (8).

					(7)

			(8)

### A Multivariate Nonlinear Mixed Effect Model (Model Specification)

Based on the multiple transformation equations between non-compartment model PK parameters and one or two compartment models, a multivariate nonlinear mixed effect model is established to estimate the population level PK parameters and their between study variances. Denote *w_jk_* as the observed *j *th non-compartment PK parameter (*j=1*,…, *J_k_*) from study *k *(*k*=*1,..,K*). Please note that not every study published all of the non-compartment parameters, hence *J_k_* varies from study to study. **β***_k_* is the study level compartment-model PK parameter vector, and *g_j _*(**β*_k_***) represents the transformation function. Because non-compartment model PK parameter,*w_jk_*, is usually published in the form of a sample mean, model (9) shows that its variance is , where is the within study variance (assumed to be homogeneous across studies), and *n_k_* is study *k* sample size

			(9)	

Model (9) also shows that the observed non-compartment model parameters, , are independent. This is a multivariate nonlinear regression model.

Study level compartment model parameter **β*_k_*** is assumed to follow multivariate normal distribution (10), in which **µ** is the population PK parameter vector and **Ω***_k_* is its general covariance matrix.

.	 (10)

The joint likelihood of population/subject parameters and their covariance is shown in equation (11).

(11)

whereis a *J×1* () observed non-compartment model PK parameter vector;is a *J×p* indicator matrix, and **X**_*k*_ is a *J_k_×p* matrix indicating the corresponding transformation function; **g**(.) is a *p×1* transformation function vector; is a study level compartment-model PK parameter vector; is a diagonal *J×J* covariance matrix for **W**,* a*nd ; is a *Kp×p* design matrix relating study-specific parameter **β** to population parameter **µ**, and **I***_k_* is an identity matrix; and is a *Kp×Kp* covariance matrix for study-specific parameter **β**.

This multivariate nonlinear mixed model (11) is different from the conventional univariate nonlinear mixed model [17] structurally in the additional design matrix **X** in front of the nonlinear function ( i.e. transformation function **g**(.)). Model (11) is a meta-analysis approach, in which sample mean non-compartment model PK parameters are formulated. Among the existing nonlinear mixed model meta-analysis literatures, some dealt with the subject-level data from multiple studies [18,19]; the others dealt with sample mean drug concentration data [20,21]; and none of them discussed the meta-analysis on summarized PK parameters through the non-compartment model.

### A Multivariate Nonlinear Mixed Effect Model (Estimation and Inference)

As a conditional first order linearization approach provides the least biased estimate in estimating the PK parameter with comparable efficiency [22,23]), it is chosen as the estimation approach for this multivariate nonlinear mixed model. This conditional first order linearization approach was firstly introduced by Lindstrom and Bates [24]. We revise their derivation based on our special meta-analysis multivariate nonlinear mixed model (11). This two-step estimation scheme is described as following.

Step 1: given the current estimate of variance componentand, minimize the following objective function, *L_1_*, with respect to (**β, µ**).

.	(12)

Computationally, minimizing *L_1_* on (**β, µ**) is an iterative process. Within each iteration, a linearization is applied to **Xg(β)** with respect to **β**, and a linear mixed model (13) is fitted [24].

		(13)

Parameters (**μ, b, β**)’s estimates and their covariance are

			(14)

			(15)

Step 2: given the current estimate,, minimize the following objective function, *L_2_* , with respect to **θ**, which is the variance component parameter vector in (**Ω, Σ**), and it is of dimension *q*.

		(16)

This *L_2_* likelihood function is the restricted maximum likelihood for variance component estimates. The scores and the elements of information matrix for **θ** are defined in (17).

		(17)

Hence, **θ** can be estimated through an iterative Fisher algorithm. An alternative derivation of this two-step first order linearization is through a second order Laplace’s approximation [25-27].

## Results

### Midazolam Non-Compartment Model Parameters to Compartment Model Parameters Transformation Data Analysis

After extensive literature search, 10 midazolam pharmacokinetics studies were identified, and their published non-compartment PK parameters are reported in Table 1. (*C_max,_ AUC, T_1/2,slow_*) were reported with high frequencies, i.e. 8 to 10 out 10 publications. *T_1/2,fast_* was published only twice. Both *V_d_* and *CL_iv_* were published 5 to 6 times.

**Table 1:** Summary of Published Non-Compartment Model Midazolam Pharmacokinetics Parameters

Non-Compartment PK Parameters	Reported	Missed
**C_max_**	9	1
**AUC**	10	0
**T_max_**	7	3
**T_1/2,fast_**	2	8
**T_1/2,slow_**	8	2
**V_d_**	5	5
**CL_iv_**	4	6

There are totally 10 studies available from publications. This table shows the number of reported and missed records for the sample means of non-compartment PK parameters among those 10 studies.

A multivariate nonlinear mixed effect model is fitted to these published non-compartment PK parameters to estimate their compartment model PK parameters. The NONMEM code is reported in Appendix I. In this meta-analysis, between study variances are assumed for (*V_1,_ k_a,_ k _e_*). (*k_12,_ k_21_*) were assumed to be the fixed effects across different studies without random effects, because only two papers published the MDZ distribution information, i.e. *T_1/2,fast_*. All of the non-compartment model parameters were log-transformed. They were assumed to have the same within study variance in log-scale (i.e. same coefficient of variance in the raw scale). All of the compartment model PK parameters were also log-transformed, and their between study standard deviations can be interpreted as coefficient of variance in raw scale.

**Figure 1:**
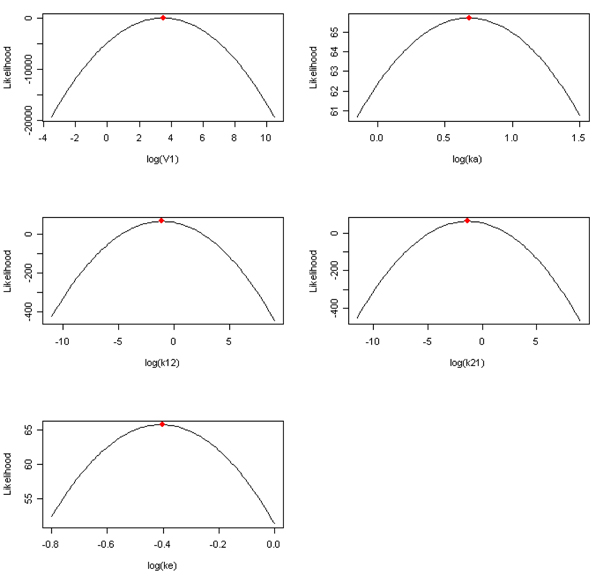
Convergence plots for five two-compartment midazolam pharmacokinetics parameters. The x-axes are log-transformed PK parameters, and y-axes are the log-likelihood functions. The dots on the top represent the maximum likelihood estimates.

Figure 1 displays the convergence plots for all five compartment PK parameters (*V_1,_ k_a,_ k_12,_ k_21,_ k_e_*). The x-axes are these PK parameters’ domain, and the y-axes are the likelihood function (13). It appears that all these PK parameter estimates reach maximum likelihood, and we don’t observe any non-identifiable parameters.

Table 2 reported the PK parameter estimates*. V_1_* = 33 L, *k_a_* = 0.68 1/h, *k_12_* = 0.33 1/h, *k_21_* = 0.27 1/h, and *k_e_* = 0.67 (1/h). Please notice that *V_1_* has very small between study variances, CV= 10%; *k_a_* has high between study variance, CV = 84%; and *k_e_*’s variation is moderate, CV = 23%. On the other hand, the within-study variation of reported non-compartment PK parameters is moderate, CV = 27%.

**Table 2:** Midazolam Compartment Model Pharmacokinetics Parameter Estimates

Compartment Model PK Parameters	Non-Compartment Model to Compartment Model Transformation	
	**Fixed-Effect**		
		
	**log-scale**	**raw-scale**	Between Study CV*

*V_1_*	3.5	33.11	10%
*k_a_*	0.68	1.97	84%
*k_12_*	-1.1	0.33	-
*k_21_*	-1.32	0.27	-
*k_e_*	-0.403	0.67	23%

Within-Study CV**		27%	

These compartment model PK parameters are estimated from reported non-compartment model PK parameters. *These are between-study variances for compartment model PK parameters. **This is the within-study variance for all non-compartment PK parameters.

### Simulation Studies

#### Simulation Schemes

The primary concern of this non-compartment PK parameter transformation to compartment model PK parameter is the bias of PK parameter estimates. Two simulation studies were designed to investigate this problem. In the first simulation, every non-compartment PK parameter was observed for each study. In the second simulation, the same amount of missing data as our MDZ example was assumed to be present.

In each simulation, 1000 simulated data sets were generated. Each data set had 10 studies, and each study reported either all (*C_max,_ AUC, T_1/2,slow_*, *T_1/2,fast_* , *V_d_* , *CL_iv_*) in simulation 1, or a partial amount of (*C_max,_ AUC, T_1/2,slow_*, *T_1/2,fast_* , *V_d_* , *CL_iv_*) in simulation 2. These non-compartment model PK parameters were simulated based on the two-compartment model transformation relationship (5) and (6), their meta-analysis multivariate nonlinear mixed model (9) and (10), and MDZ PK parameter estimates and variances from Table 2.

#### Simulation Evaluation Criteria

Both fixed effect and variance components were evaluated in the simulation studies. The bias was calculated as the relative bias: abs(true-est)/est; and their 95% coverage probabilities were also reported based on model based 95% confidence interval. Coverage probabilities outside of (92.93, 97.07) were highlighted. The half-width of this interval is three times the binomial stand error, which is [(95%)(5%)/1000]^1/2^=0.6892%. Standard error was also reported based on 1000 simulation results.

#### Simulation 1 (All Reported and No Missing Data) 

Table 3 reported the simulation results. Among fixed effects, all of the relative biases are less than 1%. (*V**1**, k_a,_ k _e_*) had lower 95% coverage probabilities than (*K_12,_ K_21_*) did, because (*V_1,_ k_a,_ k _e_*) were assumed to have between study variances, but (*K_12,_ K_21_*) didn’t have. Therefore, the low 95% coverage probability was probably due to the under estimated standard error. On the other hand, the biases of between study variance estimates were between 5% and 12.8%, though their 95% CP were all around 95%.

**Table 3:** Simulation Results with No Missing Data

		Estimate					
			
		TRUE(log-scale)	Mean	SE	RelativeBias (%)	95% CP
	*V_1_*	3.5	3.505	0.110	0.14	0.89
	*k_a_*	0.68	0.680	0.115	0.02	0.87
**Fixed-Effect**	*k_e_*	-0.403	-0.397	0.112	0.75	0.89
	*k_12_*	-1.1	-1.097	0.088	0.24	0.93
	*k_21_*	-1.32	-1.322	0.053	0.15	0.99

	*V_1_*	0.09	0.083	0.045	8.05	0.97
**Between Study Variance**	*k_a_*	0.09	0.078	0.053	12.8	0.93
	*k_e_*	0.09	0.085	0.045	5.33	0.96

**Sigma^2^**		0.01	0.01	0.003	4.43	0.95

#### Simulation 2 (With Missing Data)

Table 4 reported the simulation results. Among fixed effects, all of the relative biases can be as high as 2.84% (i.e. *k_e_*). All of their 95% coverage probabilities were outside of the normal range, (92.93%, 97.07%). The low coverage of (*V_1,_ k_a,_ k _e_*) was probably due to their between subject variations; and the low coverage probability for *k_12_* and high coverage for* k_21_* were probably due to the missing data. As in the MDZ example, *T_1/2,fast_* had only 2 out of 10 papers published. On the other hand, the biases of between study variance estimates were between 3.6% and 9.8%, though their 95% CP were within (92.93%, 97.07%).

**Table 4:** Simulation Results with Missing Data

		Estimate					
			
		TRUE(log-scale)	Mean	SD	RelativeBias (%)	95% CP
	V_1_	3.5	3.494	0.129	0.17	0.92
	k_a_	0.68	0.672	0.159	1.13	0.87
**Fixed-Effect**	k_e_	-0.403	-0.389	0.141	2.84	0.90
	k_12_	-1.1	-1.09	0.172	0.59	0.84
	k_21_	-1.32	-1.323	0.070	0.19	0.99

	V1	0.09	0.081	0.052	9.82	0.98
**Between Study Variance**	ka	0.09	0.082	0.066	9.43	0.95
	ke	0.09	0.087	0.055	3.60	0.97

**Sigma^2^**		0.01	0.01	0.003	13.9	0.96

## Conclusions

This paper proposed an important approach to transform published non-compartment model pharmacokinetics parameters into compartment model PK parameters. This meta-analysis was performed with a multivariate nonlinear mixed model. A conditional first-order linearization approach was developed for statistical estimation and inference, and it was implemented in R. Using MDZ as an example, we have shown that this approach transformed 6 non-compartment model PK parameters from 10 publications into 5 compartment model PK parameters, and the conditional first order linearization approach converged to the maximum likelihood. In the follow-up simulation studies, we have shown that our meta-analysis multivariate nonlinear mixed model had little relative bias (<1%) in estimating compartment model PK parameters if all non-compartment PK parameters were reported in every study. If there existed missing non-compartment PK parameters, the relative bias of compartment model PK parameter was still small (<3%). The 95% coverage probabilities of these PK parameter estimates were usually above 85% or more. Therefore, this approach possesses adequately valid inference.

Although this paper only showed the transformation performance of non-compartment model PK parameters to two-compartment model with oral dose PK parameters, we think it is probably the most complicated case among published drug PK studies. One compartment models and two-compartment model with IV dose have simpler transformation function and less computational expense.

Sometimes, not all of the required non-compartment model PK parameters are available in the literature. Whether it is feasible to transform these data into compartment model is an interesting and important question. In this paper, MDZ was chosen as an example. Because MDZ has been a well studied probe drug, its published non-compartment model PK parameters were expected to be rich. Other rarely studied drugs may not have all these published information, and their compartment model developments from literature need further investigations.

## List of Abbreviations

AUC: area under the concentration curve; MDZ: Midazolam; PK: Pharmacokinetics.

## Competing interests

The authors declare that they have no competing interests.

## Authors’ contributions

ZW developed the theory of multivariate nonlinear mixed effect model, and run the implementation; SK developed the theory of multivariate nonlinear mixed effect model; SKQ provided the MDZ example background; JZ integrated the compartment model non-compartment model transformation formulas; LL initialized the idea, and developed the model transformation schemes, confirmed the statistical theory, and wrote the paper.

## Authors’ information

ZW is currently a Ph.D. Computer Science student in the Indiana University; SK is an assistant professor in the University of Louisville; SKQ is an assistant professor in the Indiana University; JZ is a PhD student in the University of Michigan; and LL is an association professor in the Indiana University.
